# Beyond detection: developing probabilistic hemodynamic phenotyping after a positive point-of-care pulmonary hypertension test

**DOI:** 10.3389/fdgth.2026.1955810

**Published:** 2026-09-30

**Authors:** Farhad Fathieh, Navid Nemati, Timothy Burton, Horace R. Gillins, Ian Shadforth, Shyam Ramchandani, Charles R. Bridges

**Affiliations:** 1 Analytics for Life, Toronto, ON, Canada; 2 CorVista Health, Bethesda, MD, United States

**Keywords:** clinical decision support, Gaussian mixture model, hemodynamic phenotyping, machine learning, point-of-care diagnostics, probabilistic classification, pulmonary hypertension

## Abstract

**Background:**

Pulmonary hypertension (PH) is a heterogeneous condition in which hemodynamic subtype directs management and specialist referral. PH can be identified non-invasively at the point of care by a previously developed, regulatory-cleared machine-learned diagnostic (area under the receiver operating characteristic curve 0.95, sensitivity 82%, specificity 92%), but a positive test result raises a second clinical question: which patients have pre-capillary disease and therefore warrant referral to a specialist PH center. A probabilistic post-test phenotyping model was developed to estimate hemodynamic subtype probabilities and support rule-out of pre-capillary PH among test-positive patients.

**Methods:**

Subjects with right heart catheterization (RHC)-confirmed PH [mean pulmonary artery pressure (mPAP) ≥25 mmHg] enrolled in a prospective multisite trial (NCT04031989) who tested positive on the previously validated point-of-care PH diagnostic formed the analysis cohort (*n* = 139). Hemodynamic subtypes were assigned per 2015 European Society of Cardiology/European Respiratory Society (ESC/ERS) criteria. A one-vs.-one (OvO) Gaussian mixture model (GMM) framework, combining unsupervised clustering with *post hoc* supervised assignment of subtype probabilities was applied to features derived from non-invasive orthogonal voltage gradient and photoplethysmographic signals to generate subject-level subtype probability estimates. Blinded validation in a large dataset has not yet been performed; development results are reported, supported by a small blinded independent dataset.

**Results:**

At an exemplar *post-hoc* rule-out threshold of 16% pre-capillary probability, the GMM OvO approach yielded sensitivity 86% (95% CI: 71%–95%), specificity 51% (95% CI: 41%–61%), negative predictive value 88% (95% CI: 73%–96%) at an assumed pre-capillary prevalence of 34%, and a negative likelihood ratio (LR−) of 0.27 (95% CI: 0.07–0.70) on the development cohort. Supportive out-of-sample discrimination was observed in the blinded independent cohort.

**Conclusions:**

A GMM OvO probabilistic model applied to non-invasive point-of-care signals can generate PH subtype probability estimates and may support rule-out of pre-capillary disease among true-positive patients. The model reuses signals already acquired for PH detection and can therefore be implemented as a software addition to an existing point-of-care workflow. Independent blinded validation is required to comprehensively characterize model performance.

## 1 Introduction

Pulmonary hypertension (PH) affects approximately 114 per 100,000 adults and carries a one-year standardized mortality ratio 7.2-fold above that of matched controls, with five-year mortality reaching 62% (1). Diagnosis is frequently delayed: in pulmonary arterial hypertension (PAH), mean diagnostic delay has persisted at approximately 2.5 years, with one-third of patients waiting over two years, substantially worsening outcomes (1, 2).

PH is clinically and hemodynamically heterogeneous, encompassing five World Health Organization (WHO) groups. The central distinction governing management and specialist referral is between pre-capillary and post-capillary disease. Pre-capillary PH, characterized by elevated pulmonary vascular resistance (PVR) with normal pulmonary capillary wedge pressure (PCWP), includes PAH (WHO Group 1), PH due to lung disease (WHO Group 3), and chronic thromboembolic PH (CTEPH; WHO Group 4). PAH is amenable to guideline-directed targeted therapies including endothelin receptor antagonists, phosphodiesterase-5 inhibitors, prostacyclin pathway agents (3), and the recently approved activin signaling inhibitor sotatercept (4). PH associated with interstitial lung disease (PH-ILD) may be treated with inhaled treprostinil (5), and CTEPH with surgical pulmonary endarterectomy or guanylate cyclase stimulators (6). All pre-capillary subtypes benefit from early referral to specialist PH centers. By contrast, isolated post-capillary PH, reflecting left heart disease with elevated PCWP and normal PVR, can initially be managed by the referring clinician without specialist referral (3). Combined pre- and post-capillary PH (CpcPH), in which elevated PCWP coexists with elevated PVR, shares the elevated filling pressures of post-capillary disease while also exhibiting pulmonary vascular involvement (3). PH shows sex- and age-related differences in incidence and in diagnostic delay, and access to specialist PH-center evaluation varies geographically and socioeconomically; evidence on sociodemographic disparities in hemodynamic subtype distribution specifically remains limited (7).

To address the diagnostic delay in PH, a point-of-care diagnostic was developed (8) and prospectively validated (9) to enable earlier non-invasive identification of elevated mean pulmonary artery pressure (mPAP). The device acquires orthogonal voltage gradient (OVG) and photoplethysmographic (PPG) signals at rest and applies a machine-learned algorithm to identify elevated mPAP. The 2015 European Society of Cardiology/European Respiratory Society (ESC/ERS) definition of mPAP ≥25 mmHg (10) represents the most commonly used clinical threshold, underpinning the bulk of the published PH scientific literature to date, and therefore was applied in the development and validation of the PH detection diagnostic (8, 9). The diagnostic achieved an area under the receiver operating characteristic curve of 0.95, with 82% sensitivity at mPAP ≥25 mmHg and 92% specificity using a pre-defined algorithm cutpoint in the prospective multisite validation cohort (9), received US Food and Drug Administration (FDA) clearance in 2024 (11), and has demonstrated utility in identifying PH cases where echocardiography failed to raise suspicion (12). A positive test result does not, however, specify hemodynamic subtype.

Given that post-capillary PH and CpcPH together account for approximately two-thirds of all PH (1), a substantial proportion of test-positive patients have conditions for which initial management may not require specialist PH-center referral. The ability to identify which test-positive patients have pre-capillary disease, or to exclude it with confidence, therefore has direct implications for appropriate specialist resource utilization. Misdirected referral carries costs in both directions: unnecessary referral of patients with post-capillary disease consumes finite specialist capacity, while failure to recognize pre-capillary disease perpetuates the diagnostic delay that drives its excess mortality, as the disease-modifying therapies that improve these outcomes are accessible only through specialist evaluation.

Prior non-invasive approaches to distinguishing pre- from post-capillary PH include a multimodal algorithm combining echocardiographic indices with N-terminal pro-B-type natriuretic peptide (NT-proBNP) (13) and an echocardiographic scoring system based on structural and Doppler parameters (14), both demonstrating clinical utility. These approaches rely on skilled echocardiographic acquisition and provide binary pre- vs. post-capillary classification without explicit representation of combined disease. Neither can be executed by a non-specialist at the point where PH is first suspected, which is the setting in which the referral decision is actually made.

The approach described here addresses this gap using the same non-invasive point-of-care signals already acquired by the existing PH diagnostic, requiring no additional acquisition and modeling all three hemodynamic subtypes rather than a binary classification. A Gaussian mixture model (GMM) one-vs.-one (OvO) probabilistic framework was developed for these signals to estimate subject-level subtype probabilities across all three categories and to support rule-out of pre-capillary disease among true-positive patients, in whom the hemodynamic subtype, and therefore the appropriate care pathway, is otherwise undetermined by the detection test alone. The framework consumes signals that the cleared device already records, making it realizable in the future as a software extension of a deployed digital diagnostic rather than a new test.

## 2 Materials and methods

### 2.1 Study design and participants

This exploratory secondary analysis primarily employed data from IDENTIFY-PH (NCT04031989; protocol available on ClinicalTrials.gov), a prospective multisite clinical trial enrolling subjects with new-onset cardiovascular symptoms referred for right heart catheterization (RHC) at 18 US clinical sites (9). Participants were enrolled in IDENTIFY-PH between May 2019 and May 2021. RADPH was used for blinded assessment of the present methodology, and is composed of patients who had undergone right heart catheterization within the preceding 18 months that demonstrated a mean pulmonary artery pressure of at least 30 mmHg. RADPH enrollment occurred between April and December 2019. In both studies, as hemodynamic subtype was ascertained at the index RHC, no longitudinal follow-up was applicable. Both study protocols were approved by the Western Institutional Review Board, and written informed consent was obtained from all participants prior to study participation. This analysis is reported in accordance with the Transparent Reporting of a multivariable prediction model for Individual Prognosis Or Diagnosis + Artificial Intelligence (TRIPOD + AI) reporting guideline (15); the completed checklist is provided as Supplementary Material. Hemodynamic subtype assignments were made from RHC measurements independently of model outputs. This study did not involve any intervention, and patients received usual care from their healthcare provider. Patients and members of the public were not involved in the design, conduct, reporting, or dissemination of this research.

Subjects with RHC-confirmed PH (mPAP ≥25 mmHg) were identified from IDENTIFY-PH. This analysis was restricted to the post-test phenotyping scenario: subjects who would have received a positive result from the previously validated PH diagnostic (8). Subjects with confirmed PH but a negative diagnostic result (false negatives) were excluded, as post-test phenotyping cannot occur without a positive test result. No formal sample-size calculation was undertaken; all eligible test-positive subjects from IDENTIFY-PH were included.

Hemodynamic subtypes were assigned per the 2015 ESC/ERS PH guidelines (10) using RHC-derived mPAP, PCWP, and PVR. Pre-capillary PH was defined as mPAP ≥25 mmHg, PCWP ≤15 mmHg, and PVR >3 Wood Units (WU); isolated post-capillary PH as mPAP ≥25 mmHg, PCWP >15 mmHg, and PVR ≤3 WU; and CpcPH as mPAP ≥25 mmHg, PCWP >15 mmHg, and PVR >3 WU.

### 2.2 Signal acquisition and feature extraction

Non-invasive signals in the original study were acquired at rest over approximately 3.5 min: OVG signals using seven thoracic electrodes (one reference, three bipolar in orthogonal configuration including one posterior electrode), and PPG signals via a finger clip. The OVG provides a three-dimensional representation of cardiac electrical activity similar to the Frank lead vectorcardiograph (16), differing only by lead placement. PPG waveforms were recorded as raw red and infrared signals. Signal quality was assessed at the time of acquisition (8); recordings that failed prompted reacquisition. A library of 3,298 hand-engineered features was extracted from the combined signals across time, frequency, time-frequency, and phase-space domains, incorporating spectral, scalogram, time-series, dynamical, topological, and cross-signal synchrony analyses (8, 17, 18).

### 2.3 Probabilistic model and performance evaluation

For each of the three pairwise classifiers, candidate features were assessed using three complementary criteria: two-sample *t*-test statistic, area under the receiver operating characteristic curve, and mutual information. Features identified by any of these approaches were considered, followed by redundancy screening among highly correlated features, yielding 20 features for each pairwise classifier. Feature selection used data overlapping with the modeling cohort and therefore represents a potential source of optimistic bias.

Following supervised feature selection, the two-stage GMM OvO framework (Supplementary Material Section S2) combined unsupervised GMM fitting with supervised estimation of cluster-conditional subtype probabilities. GMM fitting was implemented using GaussianMixture from scikit-learn (version 1.8.0) (19). The features were scaled using *z*-score on the development data prior to input to the GMM. Three pairwise binary classifiers were trained: (i) pre-capillary vs. post-capillary, (ii) post-capillary vs. combined, and (iii) combined vs. pre-capillary. This pairwise decomposition reduces the multi-class problem into tractable binary subproblems suited to the limited sample size (20). For each pairwise classifier, the number of mixture components (*k* = 2–4) and covariance type (diagonal, full, tied) were selected in two sequential stages, see Supplementary Material Section S3. In a first, label-independent stage, *k* and covariance type were screened using the Bayesian information criterion and silhouette score computed from the signal features alone; full and tied covariance structures were excluded *a priori* on parameter-count grounds given the pairwise sample sizes (81–102 subjects), and silhouette score was independently maximized at *k* = 2, with diagonal covariance, in all three pairwise classifiers. In a second, *post-hoc* stage, label-conditional cluster enrichment (Fisher's exact test) was assessed for the selected two-component solution and for higher-k alternatives, confirming its clinical stability relative to the alternatives considered (Supplementary Material Section S3). No explicit class-imbalance correction (resampling or class weighting) was applied; the one-vs.-one decomposition was adopted in part to keep each binary subproblem balanced. GMMs provide soft probabilistic cluster membership, advantageous in heterogeneous, small-sample settings (19). For each pairwise GMM, cluster-conditional subtype probabilities, i.e., the conditional probability of each PH subtype given cluster membership (see Supplementary Material Section S3), were estimated from the modeling cohort. Subtype labels, previously used during feature selection, were used again at this stage to estimate cluster-conditional subtype probabilities; GMM fitting itself used the selected feature matrix without subtype labels.

For each subject, every pairwise GMM produced soft cluster memberships, expressed as the posterior probability that the subject belonged to each cluster. These cluster memberships were combined with cluster-conditional subtype probabilities using Bayes’ rule to yield a posterior probability for each pairwise class comparison. The three resulting pairwise posteriors were then fused into a single three-class probability distribution using pairwise coupling (21, 22), which enforces mutual consistency and constrains the probabilities to sum to one. This produced a subject-level probability for each of the three hemodynamic subtypes (pre-capillary, post-capillary, and combined), representing the estimated likelihood of each. Full mathematical details of the pairwise GMM probability fusion are provided in Supplementary Methods S2.

Performance was characterized on the development cohort as apparent performance and may therefore be optimistic because feature selection, GMM fitting, and estimation of cluster-conditional subtype probabilities used common data. Performance for detection of pre-capillary PH was assessed using an illustrative rule-out threshold on the pre-capillary probability: threshold-based statistics, namely sensitivity, specificity, negative predictive value (NPV), and the negative likelihood ratio (LR−), were calculated.

Formal algorithmic fairness analyses (subgroup performance parity across sex, age, and race/ethnicity) were not undertaken given the limited sample size; fairness assessment across sociodemographic groups is planned for the independent blinded validation.

### 2.4 Statistical analysis

Descriptive statistics are presented as mean ± standard deviation (SD) for continuous variables and proportions for categorical variables. All subjects had complete predictor data; no imputation was required. Clopper-Pearson exact 95% confidence intervals were calculated for sensitivity and specificity; confidence intervals for NPV and LR− were derived by propagating the sensitivity and specificity confidence limits through the respective formulae. NPV was calculated at the literature-reported pre-capillary PH prevalence of 34% among PH-positive patients (1), which differs from the observed cohort proportion; the LR− can be applied to individual pre-test odds without dependence on assumed prevalence. Calibration was assessed using calibration-in-the-large, calibration slope, Brier score and LOESS-smoothed calibration curves with bootstrap 95% confidence intervals. Discrimination across the three hemodynamic phenotypes was assessed using one-vs.-rest receiver operating characteristic area under the curve (ROC-AUC) for each phenotype. Blinded assessment was performed in RADPH, an independent dataset.

## 3 Results

### 3.1 Cohort characteristics

Of 157 subjects with RHC-confirmed PH (mPAP ≥25 mmHg) identified from IDENTIFY-PH, 18 tested negative on the PH diagnostic (false negatives) and were excluded; the remaining 139 true positives formed the analysis cohort. These comprised 37 subjects with pre-capillary PH (26.6%), 58 with isolated post-capillary PH (41.7%), and 44 with CpcPH (31.7%). The 18 patients excluded because of a negative upstream diagnostic result included 3 with pre-capillary PH, yielding 40 pre-capillary PH cases in the full RHC-confirmed PH cohort.

Baseline characteristics are shown in Table 1. Mean age was similar across groups (62–65 years), as was sex distribution (38%–49% female) and body mass index (BMI; 32–34 kg/m^2^). White/Caucasian participants comprised the majority of each phenotype group (64%–78%), while 22%–34% were Black/African American; 52%–66% were current or former smokers. Hemodynamic profiles were distinct and consistent with 2015 ESC/ERS subtype definitions: mean PVR was markedly elevated in the pre-capillary (5.8 ± 2.3 WU) and combined (5.0 ± 1.7 WU) groups relative to post-capillary (1.8 ± 0.8 WU). Mean PCWP was elevated in the post-capillary (23.4 ± 6.8 mmHg) and combined (23.1 ± 5.6 mmHg) groups and normal in the pre-capillary group (10.2 ± 3.7 mmHg). Mean mPAP was highest in the combined (40.4 ± 8.7 mmHg) and pre-capillary (38.0 ± 10.3 mmHg) groups. Cardiometabolic comorbidities were common across phenotypes, including diabetes (30%–45%), hypertension (68%–85%), and hyperlipidemia (49%–67%).

**Table 1:** Patient demographic, clinical, and hemodynamic characteristics of the analysis cohort (*n* = 139).

Characteristic	Pre-capillary (*n* = 37)	Post-capillary (*n* = 58)	Combined pre- and post-capillary (*n* = 44)
Female, %	48.6	37.9	47.7
Age, years (mean ± SD)	62.5 ± 12.0	64.9 ± 12.2	62.0 ± 12.4
BMI, kg/m^2^ (mean ± SD)	31.7 ± 6.8	32.5 ± 8.8	33.5 ± 9.2
Race/Ethnicity			
White/Caucasian	78.4%	69.0%	63.6%
Black/African American	21.6%	29.3%	34.1%
Other	0.0%	1.7%	2.3%
Smoking			
Current/Former	56.8%	51.7%	65.9%
Never	43.2%	48.3%	34.1%
mPAP, mmHg (mean ± SD)	38.0 ± 10.3	32.6 ± 6.6	40.4 ± 8.7
PVR, WU (mean ± SD)	5.8 ± 2.3	1.8 ± 0.8	5.0 ± 1.7
PCWP, mmHg (mean ± SD)	10.2 ± 3.7	23.4 ± 6.8	23.1 ± 5.6
Diabetes	29.7%	44.8%	31.8%
Hypertension	67.6%	84.5%	75.0%
Hyperlipidemia	48.6%	67.2%	59.1%

BMI, body mass index; mPAP, mean pulmonary artery pressure; PCWP, pulmonary capillary wedge pressure; PVR, pulmonary vascular resistance; SD, standard deviation; WU, wood units.

### 3.2 Model output and rule-out performance

Fewer than 1% of the features selected across all three pairwise classifiers overlapped with those used in the PH detection diagnostic (Figure 1a). The GMM OvO framework (Figure 1b) generated a three-class probability vector for each subject in the modeling cohort, illustrated in the ternary plot (Figure 1c). For an example subject, estimated probabilities were 54% pre-capillary PH, 32% post-capillary PH, and 14% CpcPH, summing to 100%.

**Figure 1:**
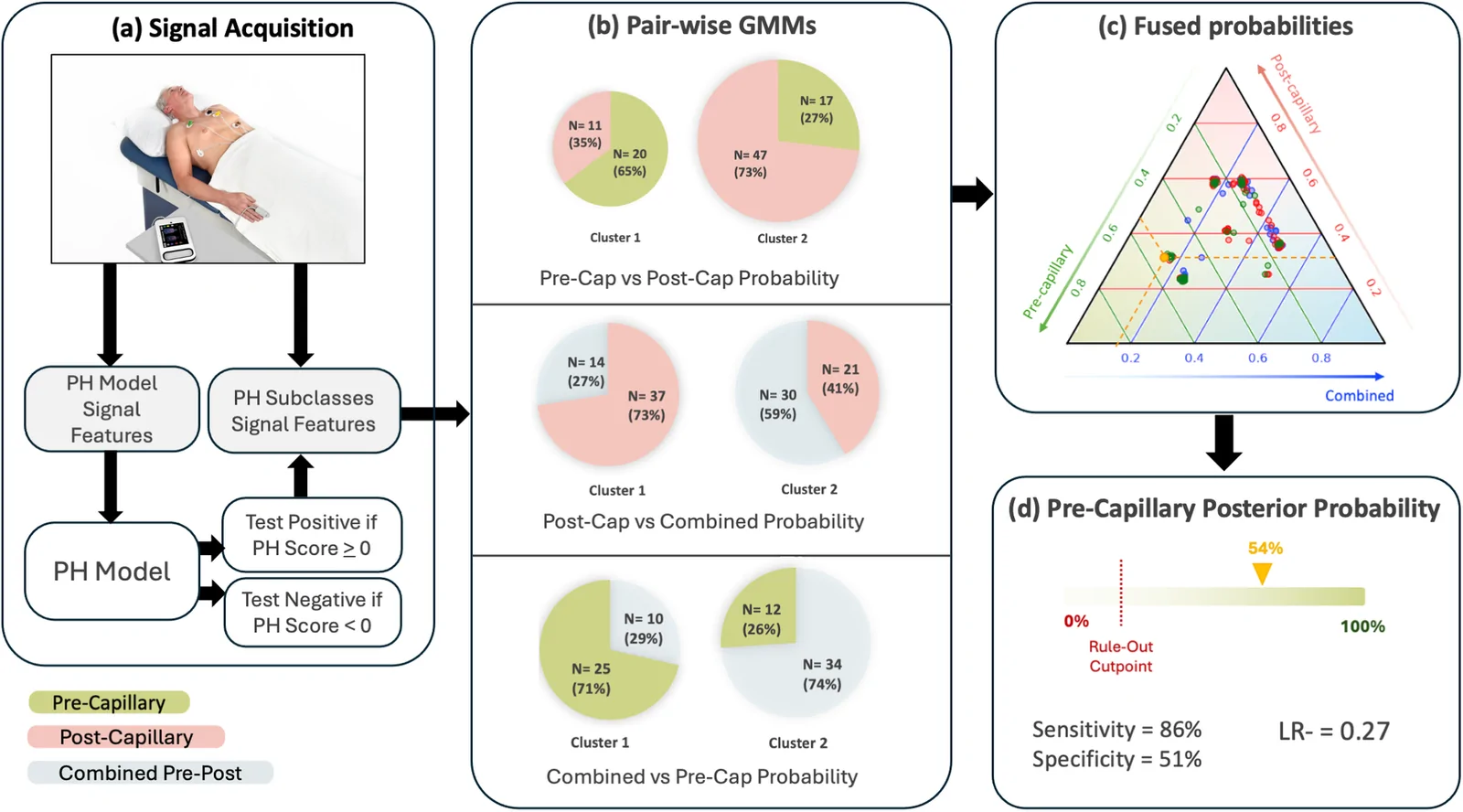
Post-test pulmonary hypertension (PH) phenotyping framework. **(a)** Signal acquisition: orthogonal voltage gradient (OVG) and photoplethysmographic (PPG) signals are acquired at rest over approximately 3.5 min, followed by feature extraction for PH phenotype classification. **(b)** Pairwise Gaussian mixture model (GMM) evaluation: given a PH-positive diagnostic result, the three pairwise GMMs are evaluated. Pie charts indicate cluster-level class composition, representing the cluster-conditional subtype probabilities estimated from the modeling cohort. **(c)** Probability fusion and ternary plot: GMM pairwise posteriors are fused using pairwise coupling to derive the estimated probability of each PH subtype, visualized as a ternary plot. Data points represent subject-level subtype probabilities from the modeling cohort and are colored according to the reference-standard RHC phenotype (pre-capillary, isolated post-capillary, or combined PH). Filled data points indicate regions with overlapping subjects. The orange data point represents an example subject; dashed projections indicate estimated probabilities of 54% pre-capillary PH, 32% post-capillary PH, and 14% combined pre- and post-capillary PH. **(d)** Rule-out interpretation: the pre-capillary posterior probability is shown on a 0%–100% scale, with the illustrative *post-hoc* rule-out cutpoint (16% pre-capillary probability) marked by the dashed line and the example subject's estimate of 54% marked in orange. Sensitivity, specificity, and the negative likelihood ratio at that cutpoint are given alongside.

The GMM OvO approach, using an illustrative rule-out pre-capillary probability threshold of 16% (Figure 1d), yielded sensitivity 86% (95% CI: 71%–95%), specificity 51% (95% CI: 41%–61%), LR− 0.27 (95% CI: 0.07–0.70), and a positive likelihood ratio of 1.76 (2 × 2 Table in Supplementary Material Section S4.1). At the literature-reported pre-capillary PH prevalence of 34% (1), the standardized NPV was 88% (95% CI: 73%–96%). Applied to an individual's pre-test odds, the LR− of 0.27 corresponds to an approximately 3.7-fold reduction in the odds of pre-capillary PH. At the observed pre-capillary PH prevalence of 26.6% in the development cohort, the NPV was 91%.

Considering the complete two-stage pathway, 37 of 40 patients with RHC-confirmed pre-capillary PH were positive on the upstream PH diagnostic, and 32 of these 37 were above the illustrative phenotyping threshold, corresponding to an end-to-end sensitivity of 80.0% (32/40; 95% CI: 64%–91%).

### 3.3 Model characterization

One-vs.-rest ROC-AUCs were 0.77 for pre-capillary PH, 0.67 for post-capillary PH, and 0.71 for combined PH, with supportive out-of-sample discrimination observed in RADPH, an independent cohort (Supplementary Material Section S4.2). In a sensitivity analysis applying the 2022 ESC/ERS hemodynamic definition, discrimination was broadly preserved, with AUCs of 0.77, 0.61, and 0.65 for pre-capillary, post-capillary, and combined PH, respectively (Supplementary Material Section S4.3). Reasonable calibration was shown in this development cohort (Supplementary Material Section S4.4), while independent evaluation remains necessary for confirmation.

## 4 Discussion

A probabilistic post-test phenotyping model was developed for patients who test positive on a validated non-invasive PH diagnostic. Applied to point-of-care signal features using a GMM OvO framework, the model generated subject-level probability estimates across three hemodynamic subtypes. The intended clinical application is to support rule-out of pre-capillary PH among test-positive patients. In this development analysis, the *post-hoc* 16% cutpoint is presented as an illustrative high-sensitivity operating point rather than a validated clinical rule-out threshold; at this cutpoint, the model achieved an NPV of 88% (95% CI: 73%–96%) and an LR− of 0.27, indicating a meaningful reduction in the likelihood of pre-capillary PH. Supportive out-of-sample discrimination was also observed in an independent cohort. Prospective validation of a pre-specified operating threshold and acceptable missed-disease rate will be required before clinical rule-out can be established.

The pre-capillary-vs.-other analysis represents one clinically motivated application of the three-class output rather than a complete referral algorithm. Since separate probabilities for isolated post-capillary PH and CpcPH are retained, a low pre-capillary probability should not be interpreted as automatic non-referral; an elevated CpcPH probability may independently support specialist assessment depending on the clinical context.

The rule-out framing is clinically relevant for pre-capillary PH because it addresses a practical limitation of PH detection alone. A positive PH diagnostic result does not distinguish the approximately one-third of test-positive patients with pre-capillary disease from the two-thirds with isolated post-capillary or combined disease. The GMM output can be used in two complementary ways. First, the three-class probability vector assigns distinct probabilities to pre-capillary, isolated post-capillary, and combined PH, representing the hemodynamic heterogeneity and providing information about the most likely phenotype. Second, for the specific clinical question of whether pre-capillary PH can be reasonably excluded, the same output can be collapsed into a pre-capillary-vs.-other paradigm and interpreted at a high-sensitivity rule-out threshold, with the corresponding LR− applied to an individual patient's pre-test probability. In this context, a low probability of pre-capillary PH may support initial management directed toward the more likely post-capillary phenotype, where clinically appropriate, rather than immediate referral of all test-positive patients to a specialist PH center. Such a result would not preclude subsequent escalation, as persistent symptoms or inadequate response to treatment for left heart disease or heart failure with preserved ejection fraction (HFpEF) would support reconsideration of CpcPH and referral for RHC.

The selected features also provide insight into the signal characteristics contributing to phenotype discrimination. The three pairwise models selected largely distinct feature sets (57 unique features among 60 selections), consistent with different signal signatures for each hemodynamic contrast. Pre-capillary vs. isolated post-capillary discrimination emphasized atrial depolarization morphology and ventricular activation–repolarization geometry, whereas post-capillary vs. combined PH was characterized predominantly by ventricular propagation and repolarization features. Pre-capillary vs. combined PH incorporated a broader mixture of ventricular and atrial morphology, beat-to-beat variability, spectral organization, and peripheral pulse characteristics. These patterns are physiologically consistent with differing contributions of pulmonary vascular and left-sided filling-pressure abnormalities, although feature-specific mechanisms were not directly evaluated (Supplementary Material Section S4.5).

### 4.1 Comparison with existing non-invasive approaches

Two prior non-invasive methods provide the closest comparison. Bonderman et al. derived a multivariable algorithm combining echocardiographic indices with NT-proBNP to exclude pre-capillary PH (13), and D'Alto et al. developed an echocardiographic scoring system to discriminate pre- from post-capillary PH (14). Both perform well, but depend on skilled echocardiographic acquisition and produce a binary pre- vs. post-capillary classification that does not explicitly represent combined disease. The threshold-based rule-out applied here shares the same clinical target, the identification or exclusion of pre-capillary PH, yet derives its estimate from point-of-care signals already acquired for PH detection and retains the full three-class probability vector, preserving the distinction between post-capillary and combined PH that a binary output discards.

### 4.2 Implementation considerations

The GMM OvO strategy is well suited to settings with substantial class overlap and limited sample sizes. The selected features have fewer than 1% overlap with those of the existing PH detection diagnostic, indicating that the two models rely on largely non-overlapping engineered features. The approach requires no additional testing beyond what is already performed for PH detection, operates at the point of care without specialist acquisition, and explicitly models all three hemodynamic subtypes.

From a deployment standpoint, this places the phenotyping estimate in a favorable position for a digital diagnostic. The signals, hardware, acquisition protocol, and clinical workflow are unchanged from those of a cleared device already in use; the phenotyping model is an additional computation on data that have already been collected and transmitted. Generating the estimate therefore adds no acquisition time, no consumables, and no retraining of staff, which matters in the non-specialist settings where PH is first suspected and where the marginal cost of an extra test is often what prevents it from being ordered. The output is a probability estimate rather than a label, which supports its use in shared decision-making and allows a clinician to weigh it against pre-test suspicion instead of accepting or rejecting a binary verdict. The underlying features are hand-engineered and physiologically interpretable, offering insight into the signal characteristics that differentiate PH subtypes, which supports clinical interpretation and future mechanistic validation.

### 4.3 Limitations

The 16% rule-out threshold was selected *post-hoc* and was not pre-specified or evaluated on a separate validation dataset; any prospective application of a threshold would require pre-specification and validation. Supervised feature selection and unsupervised GMM fitting were performed on the same dataset, and resultant performance characterization may therefore be optimistic; further, supportive out-of-sample discrimination was observed in the independent RADPH cohort, though limited by its relatively small sample size.

Subtype assignment for the primary analysis used the 2015 ESC/ERS hemodynamic thresholds (10), retained for consistency with the development and validation of the PH detection diagnostic on which this analysis is conditioned. In a sensitivity analysis applying the 2022 ESC/ERS definition (3), discrimination was broadly preserved, with modest attenuation for the post-capillary and combined phenotypes. Adjudicated WHO-group/PH etiology and treatment data were not available for this secondary analysis, limiting assessment of model performance to hemodynamic phenotypes.

Heterogeneity in performance across enrolling sites was not evaluated given limited per-site sample sizes, and formal fairness analyses across sociodemographic subgroups were not possible at this sample size. The cohort was drawn entirely from US sites in patients already referred for RHC, so the case mix is that of a pre-catheterization population rather than of unselected primary care.

Since this analysis was restricted to RHC-confirmed PH, false-positive upstream test results were not represented. Such patients do not have a ground-truth PH phenotype against which the three-class output can be evaluated. Accordingly, the reported performance applies only to true-positive PH cases and should not be interpreted as validation of the complete test-positive pathway.

## 5 Conclusion

These development-stage findings support non-invasive post-test PH phenotyping, with rule-out of pre-capillary PH as an intended clinical application. Prospective validation of a pre-specified operating threshold is required before clinical rule-out can be established. The approach provides a principled framework for post-test PH phenotyping without requiring specialist acquisition or additional testing. Applied at the point of care where PH is first detected, such triage could help direct patients with probable pre-capillary disease toward specialist evaluation sooner, addressing the diagnostic delay that contributes to their poor outcomes. The GMM OvO strategy provides a robust framework for model development in small datasets, with definitive performance characterization planned via blinded validation in an independent, enrollment-gated cohort. Such validation would also establish whether performance generalizes across the demographic and comorbidity mix encountered in routine point-of-care use; a head-to-head comparison with echocardiographic approaches in the same cohort would further contextualize the present findings.

## Data Availability

The datasets presented in this article are not readily available because they contain proprietary data; relevant de-identified subsets may be provided to academic investigators on a case-by-case basis. Requests to access the datasets should be directed to cbridges@corvista.com.
