# Supplementary material for: Beyond detection: developing probabilistic hemodynamic phenotyping after a positive point-of-care pulmonary hypertension test
Source: Front Digit Health. 2026 Sep 30;8:1955810. doi: 10.3389/fdgth.2026.1955810 (PMC13662907; doi:10.3389/fdgth.2026.1955810)
Supplement: Supplementary file 1 [file Datasheet1.docx]

Supplementary Material

**Beyond detection: developing probabilistic hemodynamic phenotyping after a positive point-of-care pulmonary hypertension test**

Farhad Fathieh, Navid Nemati, Timothy Burton, Horace R. Gillins, Ian Shadforth, Shyam Ramchandani, Charles R. Bridges

# 1 Supplementary Table 1. Completed TRIPOD+AI checklist

TRIPOD+AI reporting checklist for this analysis. Applicability codes follow the TRIPOD+AI statement: D, applicable to model development; E, applicable to model evaluation; D;E, applicable to both. Section numbers and page numbers refer to the submitted manuscript. Reference: Collins GS, Moons KGM, Dhiman P, et al. TRIPOD+AI statement: updated guidance for reporting clinical prediction models that use regression or machine learning methods. BMJ. (2024) 385:e078378.

| **Section** | **Item** | **D / E** | **Checklist item** | **Where reported** | **Comments** |
| --- | --- | --- | --- | --- | --- |
| **Title** | 1 | D;E | Identify the study as developing or evaluating the performance of a multivariable prediction model, the target population, and the outcome to be predicted. | Title page (p. 1) | Title identifies post-test PH phenotyping and the target population (test-positive patients). The abstract and Introduction state explicitly that the study reports model development. |
| **Abstract** | 2 | D;E | See TRIPOD+AI for Abstracts checklist. | Abstract (p. 2) | Abstract reported as a single paragraph structured with bold run-in labels (Background / Methods / Results / Conclusions) |
| **Introduction** | 3a | D;E | Explain the healthcare context (including whether diagnostic or prognostic) and rationale for developing or evaluating the prediction model, including references to existing models. | §1 (pp. 3–4) | Diagnostic context and rationale given; existing non-invasive models referenced (Bonderman ref 13; D'Alto ref 14). |
|  | 3b | D;E | Describe the target population and the intended purpose of the prediction model in the context of the care pathway, including its intended users. | Title page; §1 (pp. 1, 4) | Target population: patients positive on the PH detection test. Intended purpose: rule-out of pre-capillary PH to guide specialist referral at the point of care. Intended users are non-specialist clinicians at the site where PH is first suspected. |
|  | 3c | D;E | Describe any known health inequalities between sociodemographic groups. | §1 (p. 3) | Sex-, age-, geographic and socioeconomic disparities in PH described, cited to the American Thoracic Society health-disparities statement (ref 7). |
|  | 4 | D;E | Specify the study objectives, including whether the study describes the development or validation of a prediction model (or both). | Abstract (p. 2); §1 (p. 4) | Stated as development of a probabilistic post-test phenotyping model; blinded validation explicitly noted as not yet performed. |
| **Methods** | 5a | D;E | Describe the sources of data separately for development and evaluation datasets, the rationale for using these data, and representativeness. | §2.1 (p. 4) | Development used IDENTIFY-PH; supportive out-of-sample evaluation was performed in the independent RADPH cohort. |
|  | 5b | D;E | Specify the dates of the collected participant data, including start and end of participant accrual; and, if applicable, end of follow-up. | §2.1 (p. 4) | Enrollment May 2019–May 2021 stated. No longitudinal follow-up: outcome ascertained at index RHC. Dates derived from collection dates of the analysis cohort signal records. |
|  | 6a | D;E | Specify key elements of the study setting (e.g., primary/secondary care, general population) including the number and location of centres. | §2.1 (p. 4) | 18 US clinical sites; subjects with new-onset cardiovascular symptoms referred for RHC. |
|  | 6b | D;E | Describe the eligibility criteria for study participants. | §2.1 (p. 4) | RHC-confirmed PH (mPAP ≥25 mmHg) and a positive PH detection test; false negatives excluded. |
|  | 6c | D;E | Give details of any treatments received, and how they were handled during model development or evaluation, if relevant. | Not applicable | Not applicable. Phenotyping uses signals acquired at a single resting timepoint and the outcome (hemodynamic subtype) is measured at the index RHC; no interval treatment occurred between predictor and outcome measurement. |
|  | 7 | D;E | Describe any data pre-processing and quality checking, including whether this was similar across relevant sociodemographic groups. | §2.2–2.3 (p. 5) | Signal preprocessing and quality assessment are described in §2.2. Selected predictors were standardized by z-score using development-data scaling parameters before GMM fitting. |
|  | 8a | D;E | Clearly define the outcome being predicted and the time horizon, including how and when assessed, the rationale, and whether assessment is consistent across sociodemographic groups. | §2.1 (pp. 4–5) | Hemodynamic subtype (pre-capillary / post-capillary / combined) defined per 2015 ESC/ERS criteria from RHC mPAP, PCWP, PVR; assessed at index RHC. |
|  | 8b | D;E | If outcome assessment requires subjective interpretation, describe the qualifications and demographic characteristics of the outcome assessors. | §2.1 (pp. 4–5) | Not applicable: outcome derived from objective RHC hemodynamic thresholds, no subjective adjudication. |
|  | 8c | D;E | Report any actions to blind assessment of the outcome to be predicted. | §2.1 (p. 4) | Subtype assignments made from RHC measurements independently of model outputs. |
|  | 9a | D | Describe the choice of initial predictors and any pre-selection before model building. | §2.2–2.3 (p. 5) | Library of 3,298 engineered signal features; candidate features were assessed using t-test statistic, ROC-AUC and mutual information, followed by redundancy screening to yield 20 features per pairwise classifier. Feature selection used data overlapping with the modeling cohort. |
|  | 9b | D;E | Clearly define all predictors, including how and when they were measured (and any blinding). | §2.2 (p. 5) | Predictors are features derived from OVG and PPG signals acquired non-invasively at rest (~3.5 min); acquired before/independent of outcome. |
|  | 9c | D;E | If predictor measurement requires subjective interpretation, describe assessor qualifications and demographics. | §2.2 (p. 5) | Not applicable: predictors are computed automatically from acquired signals; no subjective interpretation. |
|  | 10 | D;E | Explain how study size was arrived at and justify it was sufficient; include any sample size calculation. | §2.1 (p. 4) | No formal sample-size calculation; all eligible test-positive subjects included, consistent with the exploratory development aim. |
|  | 11 | D;E | Describe how missing data were handled. Provide reasons for omitting any data. | §2.4 (p. 6) | All subjects had complete predictor data; no imputation required. |
|  | 12a | D | Describe how data were used (development/evaluation), including partitioning and sample-size considerations. | §2.3–2.4 (pp. 5–6) | IDENTIFY-PH data were used for feature selection, GMM development and development-cohort performance characterization; RADPH was used for supportive out-of-sample evaluation. |
|  | 12b | D | Describe how predictors were handled in analysis (functional form, rescaling, transformation, standardisation). | §2.3 (p. 5) | Signal preprocessing and quality assessment are described in §2.2. Selected predictors were standardized by z-score using development-data scaling parameters before GMM fitting. |
|  | 12c | D | Specify the type of model, rationale, all model-building steps including hyperparameter tuning, and method for internal validation. | §2.3 (pp. 5–6); Supplementary Material §§2–3 | The framework comprised supervised feature selection, unsupervised GMM fitting and supervised estimation of cluster-conditional subtype probabilities. Development-cohort performance is reported as apparent performance; no internal resampling was performed. |
|  | 12d | D;E | Describe if/how heterogeneity in parameter values and performance was handled and quantified across clusters (e.g., hospitals, countries). | §4.3 (p. 10) | Across-site heterogeneity not evaluated owing to limited per-site sample sizes (stated in Limitations). |
|  | 12e | D;E | Specify all measures and plots used (and rationale) to evaluate model performance and, if relevant, compare models. | §2.3–2.4 (p. 6); §3.2–3.3 (p. 7); Figure 1 legend (p. 13); Supplementary Material §§4.1–4.4 | Performance measures included sensitivity, specificity, NPV and likelihood ratios at the illustrative threshold; one-versus-rest ROC-AUCs for all three phenotypes; phenotype-specific calibration-in-the-large, calibration slope and Brier score; calibration curves; and ternary probability visualization. |
|  | 12f | E | Describe any model updating (e.g., recalibration) arising from the model evaluation. | Not applicable | Not applicable: no model updating or recalibration performed (single development analysis). |
|  | 12g | E | For model evaluation, describe how the model predictions were calculated. | §2.3 (p. 6); Supplementary Material §2 | Per-subject subtype probabilities computed via GMM posterior cluster memberships combined with cluster-conditional subtype probabilities (Bayes' rule), then fused across the three pairwise classifiers by pairwise coupling. |
|  | 13 | D;E | If class imbalance methods were used, state why and how, and any subsequent recalibration. | §2.3 (p. 5) | No explicit class-imbalance correction (no resampling or class weighting); OvO decomposition adopted in part to keep each binary subproblem balanced. |
|  | 14 | D;E | Describe any approaches used to address model fairness and their rationale. | §2.3 (p. 6); §4.3 (p. 10) | Formal fairness/subgroup-parity analyses (sex, age, race/ethnicity) not undertaken given sample size; planned for the independent blinded validation. |
|  | 15 | D | Specify the output of the prediction model; provide details and rationale for any classification and how thresholds were identified. | §2.3 (p. 6); §3.2 (p. 7); §4.3 (p. 9) | Output is a three-class probability vector (pre/post/combined). Classification illustrated via a post-hoc rule-out threshold (pre-capillary probability 16%), explicitly described as illustrative and not pre-specified or optimized on held-out data. |
|  | 16 | D;E | Identify any differences between development and evaluation data in setting, eligibility, outcome, and predictors. | §2.1 (p. 4); Supplementary Material §4.2 | Development used the IDENTIFY-PH cohort of patients with new-onset cardiovascular symptoms referred for contemporaneous RHC at 18 US sites. Supportive out-of-sample evaluation used the independent RADPH cohort, in which qualifying RHC had been performed within the preceding 18 months and demonstrated mPAP ≥30 mmHg. The same signal modalities and phenotyping framework were applied, but the cohorts differed in recruitment context and timing of the reference hemodynamic assessment. |
|  | 17 | D;E | Name the IRB/ethics committee that approved the study and describe informed consent or waiver. | §2.1 (p. 4); Ethics statement (p. 10) | Approved by the Western Institutional Review Board; written informed consent obtained from all participants prior to study participation. |
| **Open Science** | 18a | D;E | Give the source of funding and the role of the funders. | Funding (pp. 10–11) | Funded by Analytics for Life; funder role in design/data collection vs analysis/decision-to-publish/manuscript described. |
|  | 18b | D;E | Declare any conflicts of interest and financial disclosures for all authors. | Conflict of interest (p. 11) | Author employment disclosed (Analytics for Life; CorVista Health). |
|  | 18c | D;E | Indicate where the study protocol can be accessed or state that a protocol was not prepared. | §2.1 (p. 4) | The IDENTIFY-PH protocol is available on ClinicalTrials.gov (NCT04031989). A separate protocol for this exploratory secondary analysis was not prepared. |
|  | 18d | D;E | Provide registration information (register name and number) or state the study was not registered. | §2.1 (p. 4); Ethics statement (p. 10) | Registered: ClinicalTrials.gov NCT04031989. |
|  | 18e | D;E | Provide details of the availability of the study data. | Data availability statement (p. 10) | De-identified data subsets may be shared with academic investigators on a case-by-case basis. |
|  | 18f | D;E | Provide details of the availability of the analytical code. | Data availability statement (p. 10) | Model parameters and analysis code declared proprietary (i.e., not publicly available); availability statement provided. |
| **Patient & Public Involvement** | 19 | D;E | Provide details of any patient and public involvement, or state no involvement. | §2.1 (p. 4) | No patient or public involvement. |
| **Results** | 20a | D;E | Describe the flow of participants, including numbers with and without the outcome and, if applicable, follow-up time. A diagram may help. | §3.1 (p. 6) | 157 subjects with RHC-confirmed PH identified; 18 test-negative subjects excluded; 139 subjects in the analysis cohort (37 pre-capillary / 58 post-capillary / 44 combined). Counts are reported in text; no flow diagram is included. |
|  | 20b | D;E | Report characteristics overall and by source/setting, including key dates, key predictors/demographics, treatments, sample size, outcome events, follow-up, and missing data; report differences across key demographic groups. | §3.1 (pp. 6–7); Table 1 (pp. 12–13) | Table 1 reports demographics, race, smoking, diabetes, hypertension, hyperlipidemia, and hemodynamics by subtype; enrollment dates are given in §2.1. Differences across demographic groups were not formally tested, and the signal-feature predictors are not individually tabulated (high-dimensional). |
|  | 20c | E | For model evaluation, show a comparison with the development data of the distribution of important predictors. | Not applicable | This comparison was not performed. |
|  | 21 | D;E | Specify the number of participants and outcome events in each analysis. | §3.1 (p. 6); Supplementary Material §§4.2–4.3 | Development-cohort analyses included 139 subjects (37 pre-capillary, 58 isolated post-capillary, and 44 combined PH). The independent RADPH evaluation included 47 subjects (19 pre-capillary, 22 isolated post-capillary, and 6 combined PH). The 2022 ESC/ERS sensitivity analysis used the same 139-subject development cohort. |
|  | 22 | D | Provide details of the full prediction model to allow predictions in new individuals and third-party evaluation, including any access/re-use restrictions. | Data availability statement (p. 10) | Model parameters and code declared proprietary; full model specification (formula/object/API) not provided. Access restriction is stated. |
|  | 23a | D;E | Report model performance estimates with confidence intervals, including for key subgroups. | §3.2–3.3 (p. 7); Supplementary Material §§4.1, 4.2, and 4.4 | At the illustrative rule-out threshold, sensitivity was 86.5% (95% CI 71%–95%), specificity 51.0% (41%–61%), standardized NPV 88% (73%–96%) at 34% pre-capillary prevalence, and LR− 0.27 (0.07–0.70); end-to-end sensitivity was 80.0% (64%–91%). Development-cohort one-versus-rest AUCs were 0.77, 0.67, and 0.71 for pre-capillary, isolated post-capillary, and combined PH, respectively; corresponding RADPH AUCs were 0.75, 0.59, and 0.62. Phenotype-specific calibration metrics and curves are reported in Supplementary Material Section 4.4. Sociodemographic subgroup performance was not assessed because of limited sample size. |
|  | 23b | D;E | If examined, report results of any heterogeneity in model performance across clusters. | §4.3 (p. 10) | Not examined; stated in Limitations. |
|  | 24 | E | Report the results from any model updating, including the updated model and subsequent performance. | Not applicable | Not applicable: no model updating performed. |
| **Discussion** | 25 | D;E | Give an overall interpretation of the main results, including issues of fairness, in the context of objectives and previous studies. | §4 (pp. 7–10) | Interpretation provided, including comparison with Bonderman/D'Alto and the clinical rule-out rationale. Fairness not specifically discussed in interpretation (absence of fairness analysis acknowledged under item 14). |
|  | 26 | D;E | Discuss limitations and their effects on bias, statistical uncertainty, and generalizability. | §4.3 (pp. 9–10) | §4.3 addresses the post-hoc illustrative threshold, potential optimism from overlapping development data, the limited supportive independent RADPH evaluation, the 2015 versus 2022 ESC/ERS definitions, unavailable adjudicated WHO-group/etiology and treatment data, unevaluated across-site heterogeneity and sociodemographic subgroup performance, the pre-catheterization US case mix, and exclusion of upstream false-positive cases. |
|  | 27a | D | Describe how poor quality or unavailable input data should be assessed and handled when implementing the model. | §2.2 (p. 5) | §2.2: signal quality was assessed at the time of acquisition, and recordings that failed prompted reacquisition. The handling of poor-quality or unavailable input data at the phenotyping step is not specified beyond reacquisition. |
|  | 27b | D | Specify whether users must interact in handling input data or use of the model, and what expertise is required. | §4.2 (p. 9) | Partially: stated to operate at the point of care without specialist acquisition and to add no workflow change. Required user interaction/expertise not stated explicitly. |
|  | 27c | D;E | Discuss next steps for future research, with a view to applicability and generalizability. | §5 (pp. 10) | Blinded validation in an independent, enrollment-gated cohort; assessment of generalizability across the demographic and comorbidity mix; head-to-head comparison with echocardiographic approaches. |

# 2 Pairwise GMM Probability Fusion

This section provides a formal mathematical description of the pairwise Gaussian mixture model (GMM) framework introduced in Section 2.3.

## 2.1 Terminology

- **Feature vector** (*x*): The pairwise-specific set of 20 selected features for a given subject, used as input to one one-versus-one (OvO) GMM.
- **Soft cluster membership** (*qₖ*): The posterior probability, under the fitted GMM, that a subject with features x belongs to mixture component (cluster) k.
- **Cluster-conditional subtype probability** (*πₖ*): The empirical conditional probability of hemodynamic subtype A given hard cluster assignment k, estimated from the RHC-defined labels of subjects in the modeling cohort.
- **Pairwise posterior probability** (*r(A, B)*): The probability that a subject belongs to subtype A rather than B, given that the subject belongs to one of the two subtypes compared by that OvO classifier.
- **Pairwise coupling matrix** (*R*): The 3×3 matrix assembling all three pairwise posteriors (pre-capillary vs. post-capillary, post-capillary vs. combined, pre-capillary vs. combined) for a single subject.
- **Fused three-class probability vector** (*p*): The final subject-level probability distribution over the three hemodynamic subtypes (pre-capillary, post-capillary, combined).

## 2.2 Step-by-step formulation

Each of the three pairwise classifiers (pre-capillary vs. post-capillary; post-capillary vs. combined pre- and post-capillary; pre-capillary vs. combined pre- and post-capillary) is fit and combined using the same five-step procedure, applied independently per classifier and then fused across all three.

### 2.2.1 Soft cluster membership (unsupervised)

For each one-versus-one (OvO) GMM, fit using only the pairwise-selected feature vector x and without reference to hemodynamic subtype labels, each subject is assigned a soft membership over the K = 2 mixture components:

$q = \left[ q₁, q₂, \ldots, q\_K \right],\quad q\_k = P(cluster k \mid x)$	(1)

with the memberships normalized to sum to one across clusters:

$\sum_{k=1}^{K} q\_k = 1$	(2)

### 2.2.2 Cluster-conditional subtype probability (supervised step)

Using the RHC-defined hemodynamic subtype labels of subjects in the modeling cohort, each cluster k is associated with the empirical conditional probability of subtype A given (hard) membership in cluster k:

$\pi_{k} = P(class A \mid cluster k)$	(3)

For example, in the pre-capillary vs. post-capillary GMM, πₖ is the observed fraction of pre-capillary subjects among those assigned to cluster k, and 1 − πₖ is the corresponding fraction of post-capillary subjects.

### 2.2.3 Pairwise posterior probability

The subject-level soft membership q and the cluster-conditional subtype probabilities π are combined using Bayes' rule to obtain the posterior probability of class A relative to B:

$r_{A,B} = P(y = A \mid y \in\{A,B\}, x) = \sum_{k=1}^{K} q_{k} \pi_{k}$	(4)

and, by the complement rule for the two-class comparison:

$r_{B,A} = 1 - r_{A,B}$	(5)

### 2.2.4 Pairwise coupling matrix

The three pairwise posteriors (pre-capillary vs. post-capillary; post-capillary vs. combined; pre-capillary vs. combined) are assembled, for each subject, into a 3×3 coupling matrix R, with diagonal entries undefined (a class is not compared against itself) and off-diagonal entries defined as:

$R_{ij} = P(y = i \mid y \in\{i,j\}, x)$	(6)

Herein, for the given PH three subclass, the coupling matrix is defined by:

$R=\left[ \begin{matrix} 0 & r_{pre,post} & r_{pre,comb} \\ 1-r_{pre,post} & 0 & r_{post,comb} \\ 1-r_{pre,comb} & {1-r}_{post,comb} & 0 \end{matrix} \right]$	(7)

### 2.2.5 Fused three-class probability

The final subject-level probability vector $p = [p_{pre-cap}, p_{post-cap},p_{comb}]$ is obtained by solving the constrained least-squares system that enforces pairwise consistency across R^[1]^:

${min}_{p}\quad\sum_{i<j} \left( R_{ij} - \frac{p_{i}}{p_{i} + p_{j}} \right)^{2}$	(8)

subject to the non-negativity and normalization constraints:

$$p_{i} \geq0,\quad\sum_{i} p_{i} = 1$$

This step is a deterministic numerical optimization applied to the three pairwise posteriors; it introduces no additional supervision beyond that already present in π.

# 3 Hyperparameter selection for pairwise Gaussian mixture models

For each of the three pairwise classifiers (pre-capillary vs. post-capillary; post-capillary vs. combined pre- and post-capillary; pre-capillary vs. combined pre- and post-capillary), the number of mixture components (k) and covariance type were selected in two sequential stages.

In the first, label-independent stage, k (tested at 2–4) and covariance type (diagonal, full, tied) were screened using the Bayesian information criterion (BIC) and silhouette score, both computed from the signal features alone, without reference to RHC hemodynamic subtype labels. Full and tied covariance structures were excluded a priori on parameter-count grounds (Table S-3A). Given pairwise sample sizes of 81–102 subjects, the substantially larger parameter counts associated with full or tied covariance risk unstable covariance estimates that fit sampling noise in the modeling cohort rather than generalizable structure. Diagonal covariance was therefore used throughout. Among diagonal-covariance configurations, silhouette score was independently maximized at k = 2 in all three pairwise classifiers, while BIC continued to favor higher k in each case.

In the second, post-hoc stage, performed only after hyperparameters were fixed on the criteria above, label-conditional cluster enrichment was assessed for the selected configuration and for higher-k alternatives, to confirm the clinical stability of the selected solution. For each cluster, a 2×2 table (cluster membership vs. non-membership, by target subtype vs. other) was constructed and tested using Fisher's exact test; sensitivity, specificity, positive and negative likelihood ratios (LR+, LR-), and the odds ratio are reported for each cluster's association with the target subtype. These quantities describe the enrichment of the target hemodynamic subtype within each cluster and are distinct from the rule-out performance metrics reported for the final probabilistic model in the main text.

Across the higher-k alternatives, several small clusters (n = 6–12) contained zero subjects of one subtype in the corresponding 2×2 table, producing a mathematically degenerate odds ratio of 0 despite a nominally significant p-value; these estimates reflect a zero-cell artifact rather than genuine, stable class separation and are marked with an asterisk in the tables below. The selected k = 2 solution did not exhibit this artifact in any of the three pairwise classifiers and consistently produced two well-populated, significantly enriched clusters.

Table S-3A. Number of free covariance parameters by covariance type and number of components (d = 20 features).

| Covariance type | Parameters (k = 2) | Parameters (k = 3) | Parameters (k = 4) |
| --- | --- | --- | --- |
| Diagonal (k×d) | 40 | 60 | 80 |
| Tied (d(d+1)/2, shared) | 210 | 210 | 210 |
| Full (k×d(d+1)/2) | 420 | 630 | 840 |

## 3.1 GMM Pair: Pre-Capillary *vs* Post-Capillary

### 3.1.1 Hyperparameter sensitivity analysis

Table S-3B. Hyperparameter screen (BIC / silhouette, label-independent)

| k | covariance | BIC | silhouette |
| --- | --- | --- | --- |
| 3 | diag | 5001.897 | 0.134 |
| 4 | full | 5041.871 | 0.092 |
| 4 | diag | 5075.287 | 0.073 |
| 2 | diag | 5121.101 | 0.152 |
| 2 | tied | 5808.799 | 0.211 |
| 3 | full | 5810.637 | 0.108 |
| 4 | tied | 5813.638 | 0.123 |
| 3 | tied | 5836.028 | 0.098 |
| 2 | full | 6177.295 | 0.200 |

*Highlighted row: selected model (k = 2, diagonal covariance). Silhouette is maximized among diagonal-covariance configurations at k = 2; full and tied covariance were excluded a priori on parameter-count grounds (Table S-3A).*

## 3.2 Enrichment test (Post-Capillary target group)

Table S-3C. Number of clusters = 3, covariance type = diagonal

| Cluster | n_samples | Sensitivity | Specificity | LR+ | LR- | Odds Ratio | p-value |
| --- | --- | --- | --- | --- | --- | --- | --- |
| 1 | 1 | 0.000 | 0.973 | 0.000 | 1.028 | 0.000 | 0.389 |
| 2 | 36 | 0.224 | 0.378 | 0.361 | 2.050 | 0.176 | 0.000 |
| 3 | 58 | 0.776 | 0.649 | 2.208 | 0.346 | 6.391 | 0.000 |

Table S-3D. Number of clusters = 4, covariance type = full

| Cluster | n_samples | Sensitivity | Specificity | LR+ | LR- | Odds Ratio | p-value |
| --- | --- | --- | --- | --- | --- | --- | --- |
| 1 | 38 | 0.379 | 0.568 | 0.877 | 1.094 | 0.802 | 0.670 |
| 2 | 11 | 0.103 | 0.865 | 0.766 | 1.037 | 0.738 | 0.746 |
| 3 | 34 | 0.517 | 0.892 | 4.784 | 0.541 | 8.839 | 0.000 |
| 4 | 12 | 0.000 | 0.676 | 0.000* | 1.480 | 0.000* | 0.000 |

Table S-3E. Number of clusters = 4, covariance type = diagonal

| Cluster | n_samples | Sensitivity | Specificity | LR+ | LR- | Odds Ratio | p-value |
| --- | --- | --- | --- | --- | --- | --- | --- |
| 1 | 4 | 0.034 | 0.946 | 0.638 | 1.021 | 0.625 | 0.641 |
| 2 | 42 | 0.500 | 0.649 | 1.423 | 0.771 | 1.846 | 0.204 |
| 3 | 26 | 0.138 | 0.514 | 0.284 | 1.679 | 0.169 | 0.000 |
| 4 | 23 | 0.328 | 0.892 | 3.030 | 0.754 | 4.019 | 0.016 |

Table S-3F. Number of clusters = 2, covariance type = diagonal (selected model)

| Cluster | n_samples | Sensitivity | Specificity | LR+ | LR- | Odds Ratio | p-value |
| --- | --- | --- | --- | --- | --- | --- | --- |
| 1 | 31 | 0.19 | 0.459 | 0.351 | 1.764 | 0.199 | 0.001 |
| 2 | 64 | 0.81 | 0.541 | 1.764 | 0.351 | 5.027 | 0.001 |

## 3.3 GMM Pair: Post-Capillary *vs* Combined pre- and post-capillary

### 3.3.1 Hyperparameter sensitivity analysis

Table S-3G. Hyperparameter screen (BIC / silhouette, label-independent)

| k | covariance | BIC | silhouette |
| --- | --- | --- | --- |
| 3 | diag | 5677.299 | 0.018 |
| 4 | diag | 5679.827 | 0.033 |
| 2 | diag | 5772.587 | 0.074 |
| 2 | full | 5871.393 | 0.128 |
| 4 | full | 6124.640 | 0.078 |
| 3 | full | 6240.696 | 0.095 |
| 2 | tied | 6416.376 | 0.102 |
| 3 | tied | 6419.903 | 0.092 |
| 4 | tied | 6425.915 | 0.052 |

*Highlighted row: selected model (k = 2, diagonal covariance). Silhouette is maximized among diagonal-covariance configurations at k = 2; full and tied covariance were excluded a priori on parameter-count grounds (Table S-3A).*

Table S-3H. k = 3, covariance type = diagonal

| Cluster | n_samples | Sensitivity | Specificity | LR+ | LR- | Odds Ratio | p-value |
| --- | --- | --- | --- | --- | --- | --- | --- |
| 1 | 26 | 0.138 | 0.591 | 0.337 | 1.459 | 0.231 | 0.003 |
| 2 | 31 | 0.345 | 0.750 | 1.379 | 0.874 | 1.579 | 0.386 |
| 3 | 45 | 0.517 | 0.659 | 1.517 | 0.732 | 2.071 | 0.107 |

*Only 1 of 3 clusters (n = 26) reaches nominal significance (p = 0.003); clusters of n = 31 and n = 45 are not significantly enriched (p = 0.386, 0.107).*

Table S-3I. k = 4, covariance type = diagonal

| Cluster | n_samples | Sensitivity | Specificity | LR+ | LR- | Odds Ratio | p-value |
| --- | --- | --- | --- | --- | --- | --- | --- |
| 1 | 25 | 0.190 | 0.682 | 0.596 | 1.189 | 0.502 | 0.166 |
| 2 | 22 | 0.310 | 0.909 | 3.414 | 0.759 | 4.500 | 0.008 |
| 3 | 31 | 0.362 | 0.773 | 1.593 | 0.826 | 1.930 | 0.193 |
| 4 | 24 | 0.138 | 0.636 | 0.379 | 1.355 | 0.280 | 0.010 |

*Only 2 of 4 clusters reach nominal significance (p = 0.008, 0.010); clusters of n = 25 and n = 31 are not (p = 0.166, 0.193).*

Table S-3J. k = 2, covariance type = diagonal (selected model)

| Cluster | n_samples | Sensitivity | Specificity | LR+ | LR- | Odds Ratio | p-value |
| --- | --- | --- | --- | --- | --- | --- | --- |
| 1 | 51 | 0.638 | 0.682 | 2.005 | 0.531 | 3.776 | 0.003 |
| 2 | 51 | 0.362 | 0.318 | 0.531 | 2.005 | 0.265 | 0.003 |

*Both clusters are well-populated (n = 51 each) and significantly enriched (p = 0.003 each).*

## 3.4 GMM Pair: Pre-Capillary *vs* Combined pre- and post-capillary

### 3.4.1 Hyperparameter sensitivity analysis

Table S-3K. Hyperparameter screen (BIC / silhouette, label-independent)

| k | covariance | BIC | silhouette |
| --- | --- | --- | --- |
| 4 | full | 3858.896 | 0.090 |
| 4 | diag | 3868.405 | 0.061 |
| 3 | diag | 4071.680 | 0.092 |
| 2 | diag | 4234.234 | 0.161 |
| 3 | full | 4406.465 | 0.127 |
| 2 | full | 4951.010 | 0.199 |
| 4 | tied | 4963.494 | 0.098 |
| 2 | tied | 5005.417 | 0.165 |
| 3 | tied | 5020.998 | 0.148 |

*Highlighted row: selected model (k = 2, diagonal covariance). Silhouette is maximized among diagonal-covariance configurations at k = 2; full and tied covariance were excluded a priori on parameter-count grounds (Table S-3A).*

Table S-3L. k = 4, covariance type = full

| Cluster | n_samples | Sensitivity | Specificity | LR+ | LR- | Odds Ratio | p-value |
| --- | --- | --- | --- | --- | --- | --- | --- |
| 1 | 23 | 0.432 | 0.841 | 2.718 | 0.675 | 4.027 | 0.012 |
| 2 | 11 | 0.027 | 0.773 | 0.119 | 1.259 | 0.094 | 0.010 |
| 3 | 36 | 0.297 | 0.432 | 0.523 | 1.627 | 0.322 | 0.024 |
| 4 | 11 | 0.243 | 0.955 | 5.351 | 0.793 | 6.750 | 0.019 |

*All four clusters reach nominal significance in this configuration; full covariance was nonetheless excluded a priori on parameter-count grounds, independent of this outcome.*

Table S-3M. k = 4, covariance type = diagonal

| Cluster | n_samples | Sensitivity | Specificity | LR+ | LR- | Odds Ratio | p-value |
| --- | --- | --- | --- | --- | --- | --- | --- |
| 1 | 27 | 0.162 | 0.523 | 0.340 | 1.603 | 0.212 | 0.004 |
| 2 | 26 | 0.324 | 0.682 | 1.019 | 0.991 | 1.029 | 1.000 |
| 3 | 22 | 0.514 | 0.932 | 7.532 | 0.522 | 14.426 | 0.000 |
| 4 | 6 | 0.000 | 0.864 | 0.000* | 1.158 | 0.000* | 0.029 |

*Cluster 2 (n = 26) shows essentially no discrimination (odds ratio ≈ 1.03, p = 1.000).*

Table S-3N. k = 3, covariance type = diagonal

| Cluster | n_samples | Sensitivity | Specificity | LR+ | LR- | Odds Ratio | p-value |
| --- | --- | --- | --- | --- | --- | --- | --- |
| 1 | 27 | 0.595 | 0.886 | 5.232 | 0.457 | 11.440 | 0.000 |
| 2 | 12 | 0.000 | 0.727 | 0.000* | 1.375 | 0.000* | 0.000 |
| 3 | 42 | 0.405 | 0.386 | 0.661 | 1.539 | 0.429 | 0.076 |

*Cluster 3 (n = 42) is not significantly enriched (p = 0.076).*

Table S-3O. k = 2, covariance type = diagonal (selected model)

| Cluster | n_samples | Sensitivity | Specificity | LR+ | LR- | Odds Ratio | p-value |
| --- | --- | --- | --- | --- | --- | --- | --- |
| 1 | 35 | 0.676 | 0.773 | 2.973 | 0.420 | 7.083 | <0.001 |
| 2 | 46 | 0.324 | 0.227 | 0.420 | 2.973 | 0.141 | <0.001 |

*Both clusters are well-populated (n = 35, 46) and strongly, significantly enriched.*

# 4 Performance Characterization

## 4.1 2x2 Table for Pre-Capillary PH

Table S-4A

|  | Pre-Capillary PH | Other PH Phenotypes | Total |
| --- | --- | --- | --- |
| Test-Positive | 32 (TP) | 50 (FP) | 82 |
| Test-Negative | 5 (FN) | 52 (TN) | 57 |
| Total | 37 | 102 | 139 |

## 4.2 Subgroup AUCs with Additional Cohort

The table below reports one-versus-rest AUCs for each hemodynamic phenotype in the primary manuscript cohort and in an additional independent cohort from the RADPH clinical study. RADPH enrolled patients who had undergone right heart catheterization within the preceding 18 months demonstrating a mean pulmonary artery pressure of at least 30 mmHg. This therefore represents a particularly diseased population in which ground-truth hemodynamic phenotype could be determined independently and to which the phenotyping algorithm had not previously been exposed.

Only AUC point estimates are reported given the relatively small sample sizes, particularly for the combined pre- and post-capillary subgroup in the additional cohort.

Table S-4B

| Scenario | Primary Cohort | | Additional Cohort | |
| --- | --- | --- | --- | --- |
|  | Number of Subjects | AUC | Number of Subjects | AUC |
| Pre-Capillary vs Other Subgroups | Pre-Capillary N: 37  Other Subgroups N: 102 | 0.77 | Pre-Capillary N: 19  Other Subgroups N: 28 | 0.75 |
| Post-Capillary vs Other Subgroups | Post-Capillary N: 58  Other Subgroups N: 81 | 0.67 | Post-Capillary N: 22  Other Subgroups N: 25 | 0.59 |
| Combined Pre-Post vs Other Subgroups | Combined Pre-Post N: 44  Other Subgroups N: 95 | 0.71 | Combined Pre-Post N: 6  Other Subgroups N: 41 | 0.62 |

## 4.3 Performance using the 2022 ESC/ERS hemodynamic definition^[2]^

Table S-4C

| Phenotype | 2015 ESC/ERS Definition AUC | 2022 ESC/ERS Definition AUC |
| --- | --- | --- |
| Pre-Capillary PH | 0.77 | 0.77 |
| Post-Capillary PH | 0.67 | 0.61 |
| Combined Pre- Post-Capillary PH | 0.71 | 0.65 |

## 4.4 Calibration

**Calibration-in-the-large was estimated using logistic recalibration with the logit of the predicted probability included as an offset (slope fixed at 1), while calibration slope was estimated from a logistic calibration model with a free intercept and slope. LOESS calibration curves were fit to subject-level one-versus-rest outcomes using a span of 0.75, and 95% confidence bands were obtained from 1,000 subject-level bootstrap resamples.**

**Calibration was generally favorable, with mean predicted probabilities closely matching observed prevalence, calibration-in-the-large near zero, and calibration slopes near 1 across phenotypes. Modest under-dispersion was observed for pre-capillary and combined PH.**

Table S-4D

|  | **Pre-Capillary** | **Post-Capillary** | **Combined Pre- Post-Capillary** |
| --- | --- | --- | --- |
| N | 37 | 58 | 44 |
| Observed Prevalence | 26.6% | 41.7% | 31.7% |
| Mean Predicted Probability | 26.2% | 43.1% | 30.7% |
| Calibration-in-the-large | 0.024 | -0.059 | 0.046 |
| Calibration Slope | 1.20 | 0.96 | 1.36 |
| Brier Score | 0.166 | 0.227 | 0.193 |

|  |  |  |
| --- | --- | --- |

## 4.5 Feature Interpretability

Feature selection was performed separately for each pairwise comparison, permitting each GMM to identify signal characteristics relevant to its specific hemodynamic contrast. Across the three classifiers, 60 features were selected, representing 57 unique feature identifiers; only three features were shared between two classifiers and none was selected in all three. For descriptive interpretation, the selected features were grouped according to the signal characteristic they quantify: atrial activation/P-wave morphology, ventricular activation and repolarization geometry or propagation, electrical variability/time-frequency organization, peripheral pulse morphology, and respiratory or OVG–PPG coupling (Table S-4E). These groupings were applied post hoc for interpretation and were not used during feature selection or model development.

**Pre-capillary versus isolated post-capillary PH** (Table S-4F)**.** The selected feature set included substantial representation of both atrial electrical morphology and ventricular activation/repolarization geometry. Atrial descriptors included P-wave loop perimeter and planar area, terminal P-wave duration, total P-wave area, and maximal atrial-vector geometry. Ventricular descriptors included multiple spatial relationships between depolarization and repolarization vectors, ventricular loop area/perimeter, and T-wave planarity. This pattern suggests that discrimination between predominantly pre-capillary and isolated post-capillary physiology may involve electrical correlates of differing atrial and ventricular loading and remodeling. PPG and respiratory-modulation features provided additional information regarding peripheral pulsatile and cardiorespiratory behavior.

**Isolated post-capillary versus combined pre- and post-capillary PH** (Table S-4G)**.** This comparison was characterized most strongly by ventricular activation and repolarization features, including QRS and T-wave orbital-frequency descriptors, QRS geometry, ventricular-vector orientation, and repolarization timing. Because both phenotypes are characterized by elevated left-sided filling pressure, whereas CpcPH additionally includes a pre-capillary pulmonary vascular component, these features may reflect electrical consequences of the additional ventricular loading associated with CpcPH. PPG morphology and time-frequency descriptors contributed complementary peripheral pulse information.

**Pre-capillary versus combined pre- and post-capillary PH** (Table S-4H)**.** The selected set was more heterogeneous, incorporating ventricular activation/repolarization geometry, atrial depolarization descriptors, beat-to-beat cycle variability, spectral/coherence features, PPG morphology, and OVG–PPG synchronization. Since both phenotypes contain a pre-capillary component but differ principally in the presence of elevated post-capillary filling pressure, this broader signal signature may reflect the additional atrial and ventricular consequences of left-sided filling pressure in CpcPH.

These interpretations provide physiological context for the mathematically defined signal features but should not be interpreted as demonstrating feature-specific mechanisms. The present study did not directly relate individual features to chamber dimensions, ventricular function, filling pressures, or pulmonary vascular remodeling.

The following definitions are of use in the reading of tables S-4F through H. OVG denotes the orthogonal voltage gradient signal, acquired along three mutually orthogonal axes: Orth1 (X) is the left-to-right axis, Orth2 (Y) is the superior-to-inferior axis, and Orth3 (Z) is the anterior-to-posterior axis; Orth12, Orth13, and Orth23 denote two-axis projections, while Orth123 uses all three axes. PPG denotes photoplethysmography; VPG is the first derivative of the PPG waveform and APG is its second derivative. AD, VD, and VR denote atrial depolarization, ventricular depolarization, and ventricular repolarization, respectively; MVDV, IVDV, and TVDV denote maximal, initial, and terminal ventricular depolarization vectors, MVRV the maximal ventricular repolarization vector, and MADV the maximal atrial depolarization vector. CV denotes cycle variability, PSD power spectral density, MMD maximum mean discrepancy, and DUO denotes features derived from interactions between OVG and PPG signals.

Table S-4E

| **Signal-level domain** | **Pre-capillary vs post-capillary** | **Post-capillary vs combined PH** | **Pre-capillary vs combined PH** |
| --- | --- | --- | --- |
| Atrial activation / P-wave morphology | 5 | 3 | 4 |
| Ventricular activation/repolarization geometry & propagation | 10 | 11 | 8 |
| Electrical variability / spectral / time-frequency organization | 1 | 3 | 5 |
| Peripheral pulse morphology (PPG) | 2 | 3 | 2 |
| Respiratory or OVG-PPG coupling | 2 | 0 | 1 |
| Total | 20 | 20 | 20 |

Table S-4F

| Pre-capillary PH versus isolated post-capillary PH | | |
| --- | --- | --- |
| Feature identifier | Signal | Brief description |
| visual_ovg_AD_2dPerimeter_Overall_orth23 | OVG | Overall perimeter of the atrial depolarization (P-wave) loop projected onto the orth23 plane. |
| visual_ovg_MVDV_MVRV_2dAngle_orth23 | OVG | Angle between the maximal ventricular depolarization and repolarization vectors projected onto the orth23 plane. |
| visual_ovg_AD_MaxFitPlanarArea_orth123 | OVG | Area enclosed by the atrial depolarization point cloud after projection onto its best-fitting plane. |
| visual_ovg_VR_2dPerimeter_Quad3_orth23 | OVG | Perimeter of the ventricular repolarization (T-wave) loop within quadrant 3 of the orth23 projection. |
| visual_ppg_peakVPG_Elevation_IR | PPG | Elevation of the peak-VPG landmark vector above the PPG-VPG plane in standardized PPG/VPG/APG space (infrared channel). |
| visual_ovg_MVDV_MVRV_3dAngle_orth123 | OVG | Three-dimensional angle between the maximal ventricular depolarization and repolarization vectors. |
| phys_ovg_dist_terminalSegment_orth1 | OVG | Duration of the terminal P-wave segment in orth1, with segment boundaries determined by waveform morphology. |
| resp_duo_dAmMMDStd_orth123 | OVG + PPG | Standard deviation across windows of the spectral discrepancy (MMD) between OVG amplitude modulation and the reconstructed respiratory proxy. |
| rwp_ovg_T_max_Planarity_sub3_orth123 | OVG | Maximum planarity descriptor of the three-dimensional T-wave trajectory in subband 3. |
| resp_duo_dFmEntKurt_red_infrared | PPG | Kurtosis across windows of relative entropy between a uniform reference distribution and the normalized PPG frequency-modulation spectrum. |
| visual_ovg_MVDV_TVDV_cos2dAngle_orth23 | OVG | Cosine of the angle between maximal and terminal ventricular depolarization vectors projected onto the orth23 plane. |
| visual_ovg_IVDV_Vorticity_orth123 | OVG | Vorticity (curl-magnitude) descriptor of the initial ventricular depolarization vector in three-dimensional OVG signal space. |
| visual_ovg_MADV_Vorticity_orth123 | OVG | Vorticity (curl-magnitude) descriptor of the maximal atrial depolarization vector in three-dimensional OVG signal space. |
| visual_ovg_MVDV_MVRV_2dAngle_orth12 | OVG | Angle between the maximal ventricular depolarization and repolarization vectors projected onto the orth12 plane. |
| wt_ovg_CohpowerCentroid_orth13 | OVG | Power-centroid descriptor of high-coherence regions in the wavelet-coherence representation of orth1 and orth3. |
| phys_ovg_totalArea_orth3 | OVG | Total P-wave area in orth3, combining the morphology-defined first and second waveform portions. |
| rwp_ovg_T_minMaxRatio_Planarity_sub7_orth123 | OVG | Minimum-to-maximum ratio of the planarity descriptor of the three-dimensional T-wave trajectory in subband 7. |
| visual_ppg_minAPG_cos2DAngle_VPGAPG_infrared | PPG | Cosine of the minimum-APG landmark vector angle relative to the VPG axis in the VPG-APG projection (infrared channel). |
| visual_ovg_VR_Max2dAreaQuadNum_orth13 | OVG | Identifier of the quadrant containing the largest projected T-wave-loop area in the orth13 plane. |
| visual_ovg_VD_2dArea_Quad2_orth13 | OVG | Area of the ventricular depolarization (QRS) loop within quadrant 2 of the orth13 projection. |

Table S-4G

| Isolated post-capillary PH versus combined pre- and post-capillary PH | | |
| --- | --- | --- |
| Feature identifier | Signal | Brief description |
| waveDuration_V1 | OVG | P-wave onset-to-offset duration descriptor in the V1-labelled representation. |
| visual_ppg_peakVPG_sin2DAngle_PPGVPG_infrared | PPG | Sine of the peak-VPG landmark vector angle relative to the PPG axis in the PPG-VPG projection (infrared channel). |
| visual_ovg_VD_2dPerimeter_Quad4_orth23 | OVG | Perimeter of the QRS loop within quadrant 4 of the orth23 projection. |
| visual_ovg_VD_SurfaceArea_AlphaShape_Octant5_orth123 | OVG | Alpha-shape surface area of the three-dimensional QRS point cloud within octant 5. |
| phys_ovg_dower_ampIntialPeak_V1 | OVG | Initial P-wave peak amplitude relative to the onset-offset baseline in Dower-derived lead V1. |
| visual_ovg_MVRV_2DAngle_orth23 | OVG | Angle of the maximal ventricular repolarization vector relative to the leading axis of the orth23 projection. |
| rwp_ovg_T_min_OrbitalFrequency_sub14_orth123 | OVG | Minimum orbital-frequency descriptor (three-dimensional vector rotation rate) of the T-wave trajectory in subband 14. |
| dwp_ovg_QRS_max_OrbitalFrequency_sub14_orth123 | OVG | Maximum orbital-frequency descriptor (three-dimensional vector rotation rate) of the QRS trajectory in subband 14. |
| psd_ovg_powerRatioSignal_orth2 | OVG | Ratio of orth2 spectral power to total OVG signal power. |
| repo_ovg_tpTeNormalized_orth2 | OVG | Normalized T-peak-to-T-end descriptor in orth2. |
| wt_ovg_PwaveDist_peakDispPSDnanmedian_orth2 | OVG | Median P-wave spectral peak-dispersion descriptor in orth2, based on spectral peak amplitude divided by peak width. |
| lvedpvisual_ovg_meanQRS_cos_AngleSP_orth23 | OVG | Cosine of the mean-QRS angular descriptor in the sagittal (orth23) projection. |
| wt_ppg_vpg_frequencyCentroid_nanmedian_red_IR | PPG | Median frequency-centroid descriptor of VPG wavelet-spectrum regions, using the red and infrared PPG inputs. |
| dwp_ovg_QRS_min_OrbitalFrequency_sub9_orth123 | OVG | Minimum orbital-frequency descriptor (three-dimensional vector rotation rate) of the QRS trajectory in subband 9. |
| visual_ppg_peakAPG_sin2DAngle_VPGAPG_infrared | PPG | Sine of the peak-APG landmark vector angle relative to the VPG axis in the VPG-APG projection (infrared channel). |
| cv_ovg_CV_Perimeter_orth23 | OVG | Perimeter of the alpha-shape cycle-variability point cloud projected onto the orth23 plane. |
| psd_ovg_coherence_sum_orth23 | OVG | Cumulative spectral coherence between orth2 and orth3. |
| visual_ovg_IVDV_2DAngle_orth23 | OVG | Angle of the initial ventricular depolarization vector relative to the leading axis of the orth23 projection. |
| rwp_ovg_T_median_OrbitalFrequency_sub2_orth123 | OVG | Median orbital-frequency descriptor (three-dimensional vector rotation rate) of the T-wave trajectory in subband 2. |
| lvedpvisual_ovg_detlaInitialTerminalQRSDuration_orth123 | OVG | Difference descriptor for initial and terminal QRS durations derived from three-dimensional OVG. |

Table S-4H

| C. Pre-capillary PH versus combined pre- and post-capillary PH | | |
| --- | --- | --- |
| Feature identifier | Signal | Brief description |
| visual_ovg_VD_Max2dAreaQuadNum_orth12 | OVG | Identifier of the quadrant containing the largest projected QRS-loop area in the orth12 plane. |
| cv_ovg_CV_Perimeter_orth23 | OVG | Perimeter of the alpha-shape cycle-variability point cloud projected onto the orth23 plane. |
| cv_ovg_CV_relaive_orth1 | OVG | Relative contribution of orth1 to cycle variability: CV(orth1) divided by the sum of CV across all three OVG channels. |
| psd_ovg_ssr_orth23 | OVG | Sum of squared residuals from a lognormal fit to the orth2-orth3 coherence density distribution. |
| linear_ovg_std_ventricularDepolarRepolarPeakDelay_orth2 | OVG | Standard-deviation descriptor of the delay between ventricular depolarization and repolarization peaks in orth2. |
| visual_ovg_VR_2dPerimeter_Quad3_orth23 | OVG | Perimeter of the ventricular repolarization (T-wave) loop within quadrant 3 of the orth23 projection. |
| wt_ovg_CohpowerCentroid_orth23 | OVG | Power-centroid descriptor of high-coherence regions in the wavelet-coherence representation of orth2 and orth3. |
| visual_ovg_MADV_Vorticity_orth123 | OVG | Vorticity (curl-magnitude) descriptor of the maximal atrial depolarization vector in three-dimensional OVG signal space. |
| lvedpvisual_ovg_meanQRS_T_AngleSP_orth23 | OVG | Mean-QRS-to-T angular descriptor in the sagittal (orth23) projection. |
| visual_ovg_VR_2dArea_Quad3_orth23 | OVG | Area of the T-wave loop within quadrant 3 of the orth23 projection. |
| sync_duo_dYMode2_red_infrared_orth2 | OVG + PPG | Mode of the Py2 distribution in Poincare Map 1: orth2 amplitudes sampled at PPG crossover events. |
| lvedpvisual_ovg_maxQRS_sin_AngleSP_orth23 | OVG | Sine of the maximal-QRS angular descriptor in the sagittal (orth23) projection. |
| visual_ovg_VR_2dPerimeter_Quad1_orth23 | OVG | Perimeter of the T-wave loop within quadrant 1 of the orth23 projection. |
| wt_ovg_QRSwave_timeCentroidmedian_orth1 | OVG | Median temporal centroid of thresholded QRS wavelet-spectrum regions in orth1. |
| wt_ovg_Pwave_eccentricityiqr_orth2 | OVG | Interquartile range of the eccentricity descriptor for P-wave time-frequency regions in orth2. |
| dist_terminalSegment_I | OVG | Terminal P-wave segment-duration descriptor in the lead-I-labelled representation. |
| visual_ppg_peakVPG_2DAngle_PPGAPG_infrared | PPG | Angle of the peak-VPG landmark vector relative to the PPG axis in the PPG-APG projection (infrared channel). |
| visual_ovg_VD_2dPerimeter_Quad1_orth13 | OVG | Perimeter of the QRS loop within quadrant 1 of the orth13 projection. |
| visual_ovg_AD_AverageVelocity_orth123 | OVG | Average distance between consecutive points of the three-dimensional atrial depolarization trajectory at a fixed sampling rate. |
| linear_ppg_APG_std_min_red | PPG | Standard deviation of APG minimum amplitudes across detected cycles in the red PPG channel. |

1. Wu TF, Lin CJ, Weng RC. Probability estimates for multi-class classification by pairwise coupling. J Mach Learn Res. (2004) 5:975–1005.
2. Humbert, M., Kovacs, G., Hoeper, M.M., Badagliacca, R., Berger, R.M., Brida, M., Carlsen, J., Coats, A.J., Escribano-Subias, P., Ferrari, P. and Ferreira, D.S., 2022. 2022 ESC/ERS Guidelines for the diagnosis and treatment of pulmonary hypertension: Developed by the task force for the diagnosis and treatment of pulmonary hypertension of the European Society of Cardiology (ESC) and the European Respiratory Society (ERS).). *European heart journal*, *43*(38), pp.3618-3731.
